# Supplementary material for: Genetic interactions contribute less than additive effects to quantitative trait variation in yeast
Source: Nat Commun. 2015 Nov 5;6:8712. doi: 10.1038/ncomms9712 (PMC4635962; doi:10.1038/ncomms9712)
Supplement: Supplementary Information — Supplementary Figures 1-5 [file ncomms9712-s1.pdf]

Supplementary Figures

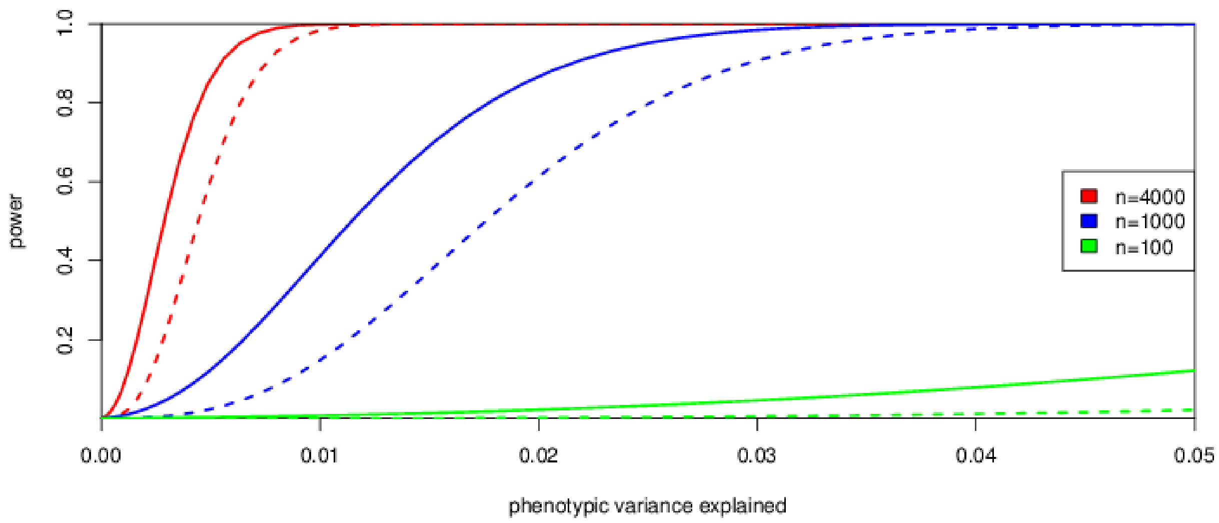

Figure S1 .

Curves illustrating statistical power are shown for mapping populations of 4000 (red), 1000 (blue), and 100 (green) segregants at a genome-wide significance threshold. The solid curves correspond to power for additive QTL and the dashed curve corresponds to power for QTL-QTL interactions.

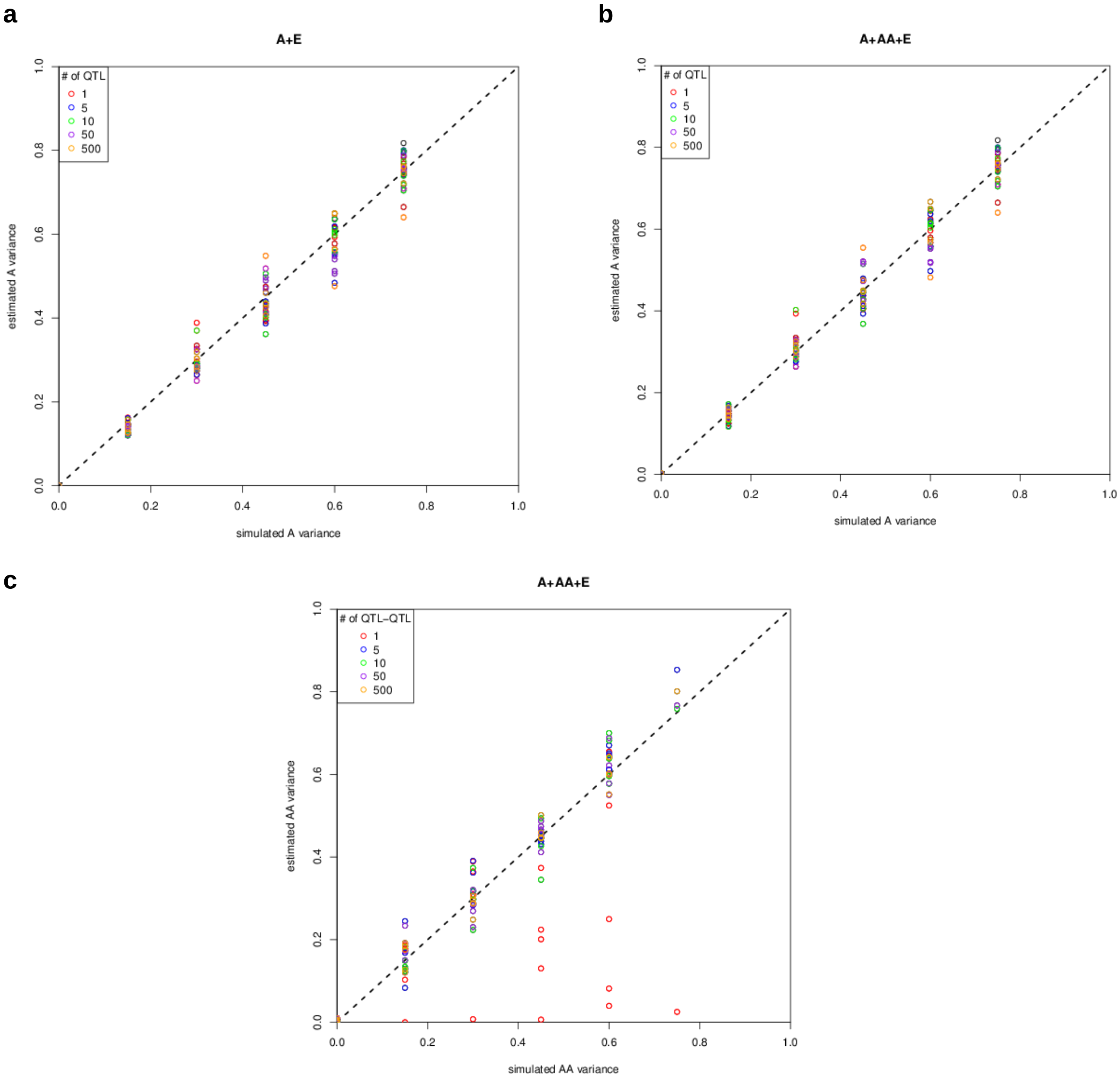

Figure S2.

The amount of simulated variance (X-axis) is plotted against whole genome based variance component estimates (Y-axis). Each point represents a particular simulation with a specific genetic architecture. Colors indicate the number of QTL(a and b) or QTL-QTL interactions (c) simulated per simulated trait. a) Estimate of the additive variance component with A+E model. b) Estimate of the additive variance component with an A+AA+E model. c) Estimate of the interaction variance (AA) component with an A+AA+E model. Simulations include a range of QTL with QTL-QTL architectures ranging from 0 to 500 for each simulated trait, adding up to a total contribution of additive (a and b) or interaction variance (c) indicated on the X-axis. Simulations include interactions, even for the A+E model. The dotted black line represents (estimated = stimulated variance) and is shown as a visual guide.

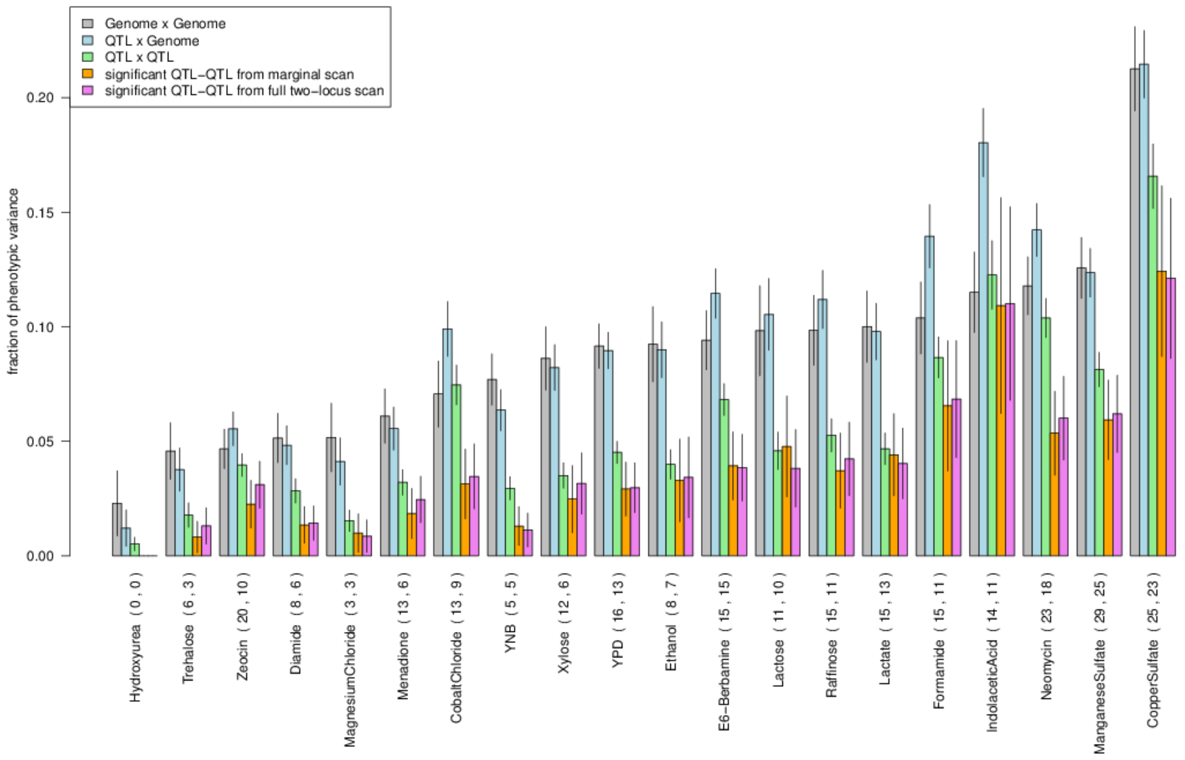

Figure S3.

For each trait, juxtaposed barplots of the two-way interaction variance captured with all markers (genome x genome) (grey), significant additive QTL by all markers (QTL x genome) (blue), additive QTL by additive QTL (QTL x QTL) (green), significant QTL-QTL interactions detected from the marginal scan (orange), and significant QTL-QTL interactions from the exhaustive two-dimensional scan (purple) are shown. Error bars show +/- s.e.

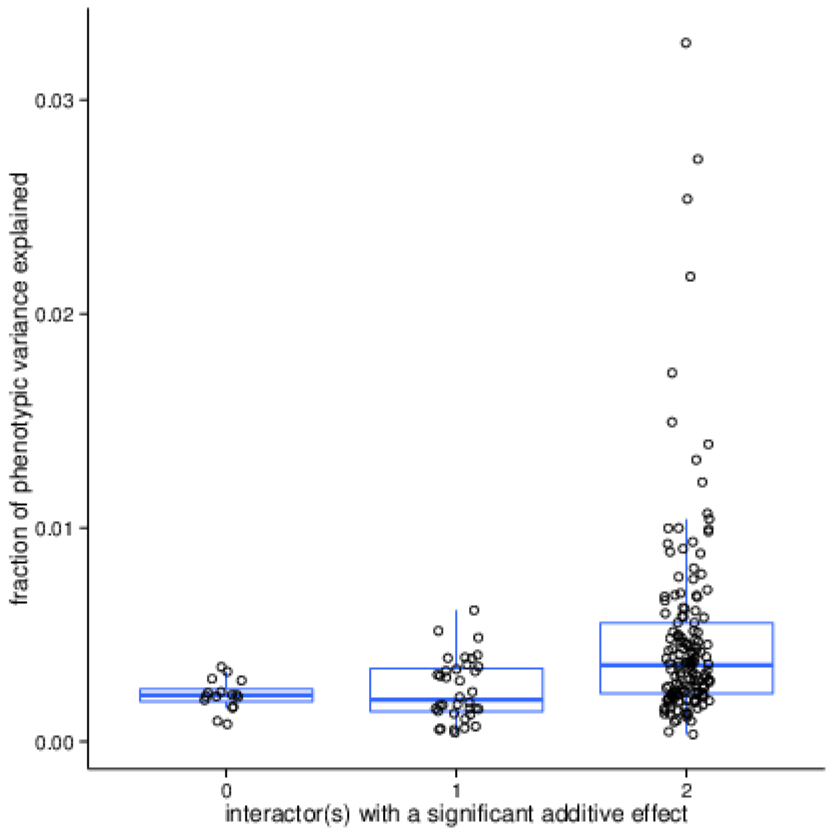

Figure S4.

The fraction of phenotypic variance explained by individual significant QTL-QTL interactions from the exhaustive two-dimensional scan aggregated across all traits and grouped by whether 0, 1, or 2 of the interacting partners of the QTL-QTL interaction also have significant additive effects.

Figure S5.

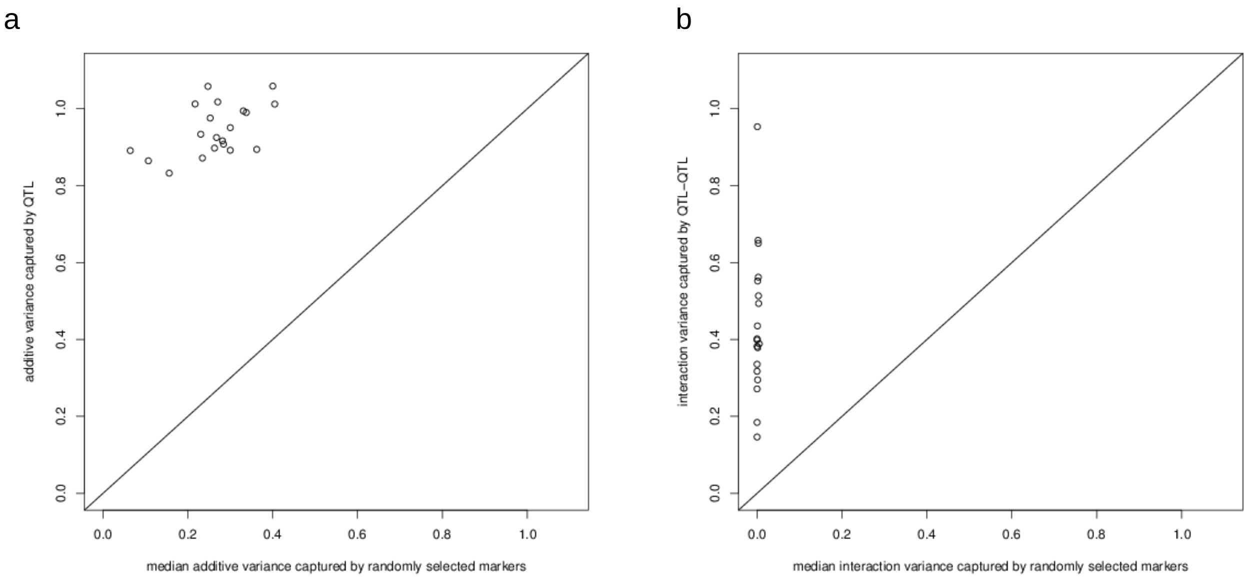

The median additive variance captured from N randomly selected markers (where N is the number of significant detected QTL for that trait) (X-axis) is plotted against the variance captured by QTL (Y-axis). The diagonal line represents (variance captured by detected QTL = variance captured due to background linkage effects) and is shown as a visual guide. B) The median interaction variance captured from N randomly selected pairs of markers (where N is the number of significant detected QTL-QTL pairs for that trait) (X-axis) is plotted against the variance captured by QTL-QTL pairs (Y-axis). The diagonal line represents (variance captured by detected QTL-QTL pairs = variance captured due to background linkage effects) and is shown as a visual guide.
